# Functional and anatomical correlates of word-, sentence-, and discourse-level integration in sign language

**DOI:** 10.3389/fnhum.2013.00681

**Published:** 2013-10-22

**Authors:** Tomoo Inubushi, Kuniyoshi L. Sakai

**Affiliations:** ^1^Department of Basic Science, Graduate School of Arts and Sciences, The University of TokyoTokyo, Japan; ^2^Japan Society for the Promotion of ScienceTokyo, Japan; ^3^CREST, Japan Science and Technology AgencyTokyo, Japan

**Keywords:** cerebral cortex, hemispheric lateralization, fMRI, frontal lobe, neuroimaging

## Abstract

In both vocal and sign languages, we can distinguish word-, sentence-, and discourse-level integration in terms of *hierarchical* processes, which integrate various elements into another higher level of constructs. In the present study, we used magnetic resonance imaging and voxel-based morphometry (VBM) to test three language tasks in Japanese Sign Language (JSL): word-level (Word), sentence-level (Sent), and discourse-level (Disc) decision tasks. We analyzed cortical activity and gray matter (GM) volumes of Deaf signers, and clarified three major points. First, we found that the activated regions in the frontal language areas gradually expanded in the dorso-ventral axis, corresponding to a difference in linguistic units for the three tasks. Moreover, the activations in each region of the frontal language areas were incrementally modulated with the level of linguistic integration. These dual mechanisms of the frontal language areas may reflect a basic organization principle of hierarchically integrating linguistic information. Secondly, activations in the lateral premotor cortex and inferior frontal gyrus were left-lateralized. Direct comparisons among the language tasks exhibited more focal activation in these regions, suggesting their functional localization. Thirdly, we found significantly positive correlations between individual task performances and GM volumes in localized regions, even when the ages of acquisition (AOAs) of JSL and Japanese were factored out. More specifically, correlations with the performances of the Word and Sent tasks were found in the left precentral/postcentral gyrus and insula, respectively, while correlations with those of the Disc task were found in the left ventral inferior frontal gyrus and precuneus. The unification of functional and anatomical studies would thus be fruitful for understanding human language systems from the aspects of both universality and individuality.

## Introduction

All human languages involve various elements at different levels of hierarchical linguistic processing (Chomsky, 1995; Jackendoff, 2002). Indeed, multiple phonemes or morphemes are combined into single words through word-level integration; multiple content words and function words are merged into single sentences through sentence-level integration, and multiple sentences are further incorporated into discourses through discourse-level integration. While higher-level elements integrate linguistic information from lower-level elements, associated meanings and contextual information emerge simultaneously. It has been proposed that unification processes of phonological, syntactic, and semantic elements are gradually represented from the ventral part of the left lateral premotor cortex [L. LPMC, the lateral side of Brodmann's areas (BAs) 6/8] to the pars orbitalis of the left inferior frontal gyrus (L. F3O, BA 47) with a *caudo-rostral* gradient (Hagoort, 2005; Uddén and Bahlmann, 2012). In contrast, we have proposed that syntax and sentence comprehension are organized in the *dorso-ventral* axis of the left lateral side of BAs 6/8, 44/45, and 47 (Sakai, 2005). Consistent with this latter possibility, previous neuroimaging studies have reported selective activation in the L. LPMC and/or pars opercularis and triangularis of the left inferior frontal gyrus (L. F3op/F3t, BAs 44/45) for syntactic processing (Stromswold et al., 1996; Dapretto and Bookheimer, 1999; Embick et al., 2000; Indefrey et al., 2001; Hashimoto and Sakai, 2002; Sakai et al., 2002; Friederici et al., 2003; Musso et al., 2003; Ben-Shachar et al., 2004; Sahin et al., 2006; Iijima et al., 2009; Inubushi et al., 2012), and in the L. F3O for sentence comprehension (Dapretto and Bookheimer, 1999; Homae et al., 2002, 2003; Sakai et al., 2005). Here we define the *frontal language areas* as the regions consisting of the L. LPMC, L. F3op/F3t, and L. F3O. The goal of the present study was to determine the functional organization of these frontal language areas. Although linguistic processes are localized in different regions according to these postulates, there has been no direct evidence regarding *how* these multiple regions are organized. Indeed, it is unknown whether each specific region within the frontal language areas is overactivated, unchanged, or underactivated at higher levels of linguistic integration, when compared with lower levels. Furthermore, if the frontal language areas play fundamental roles in various linguistic processes, then the functional organization of these regions should be independent of input modalities, including speech sounds, letters, and signs. We predicted that different levels of integration would be associated with increased activations in the dorso-ventral axis of the frontal language areas.

**Figure 1:**
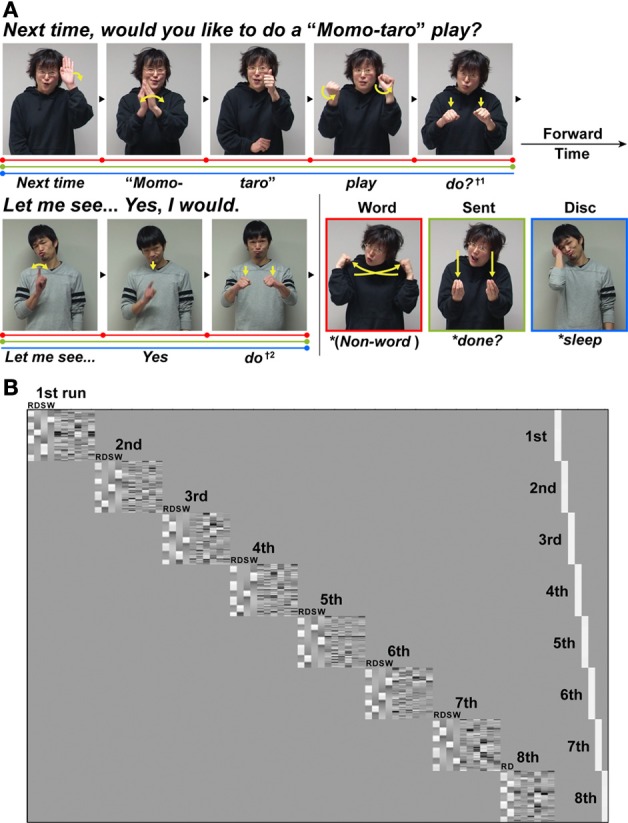
**An experimental paradigm with language tasks in Japanese Sign Language (JSL). (A)** There were three language tasks: a word-level decision (Word), a sentence-level decision (Sent), and a discourse-level decision (Disc) task. The stimuli used in the Disc task were a long dialog articulated by two signers who were taking turns (see Table 1). In both the Word and Sent tasks, the sentences from this dialog were presented in a randomized order. In these three language tasks, some words or phrases were replaced with anomalous probes (indicated by asterisks before the translated words in the figure), to which the participants were asked to respond by pressing a button. For example, in the Word task, the word “*do?*” (denoted by †1) articulated by the first signer was replaced by a pseudo-sign (the picture with a red border). In the Sent task, the word “*do?*” was replaced by a syntactically anomalous JSL sign “*done?*” (the picture with a light green border) as a perfective aspect marker expressing the past tense. In the Disc task, the word “*do*” (denoted by †2) articulated by the second signer was replaced by a contextually anomalous sign “*sleep*” (the picture with a blue border). The linguistic units for these three tasks are shown in color bars separated by dots: Word (red), Sent (light green), and Disc (blue). **(B)** The general linear model design matrix for one typical participant. Run-specific predictors are shown in the right-hand columns (denoted as 1st–8th) that model differences among individually averaged activations in eight runs. For each of the left-hand columns representing the first to seventh runs, the regressors of the R, Disc (D), Sent (S), and Word (W) tasks (convolved with a hemodynamic function), as well as the realignment parameters of three translations and three rotations obtained from preprocessing (i.e., due to head movements), were included in the design matrix in this order. For the eighth run, the regressors of the R and Disc tasks, as well as the six realignment parameters, were included in the design matrix.

In the present study, we used the Japanese Sign Language (JSL). It might be thought that a sign language is a unique or atypical language, because it is used only in a visual mode without auditory representations for most words. It is true that Deaf (we follow the recent trend in capitalizing this term to refer to a cultural group) participants almost exclusively rely on visual information for language processing throughout their lives, which is not the case for individuals with normal hearing. However, sign languages share the same basic properties of word-, sentence-, and discourse-level processes with vocal languages (Sandler and Lillo-Martin, 2006), which can be easily illustrated by the JSL sentences we used (Figure 1A, Table 1). On the other hand, we included some children (seven participants younger than 19 years old), and recruited disproportionate numbers of females (20 females out of 28 participants). Moreover, the Deaf participants showed the individual variability in JSL proficiency, which has been primarily due to limited opportunities for communication and education in JSL and the written Japanese (JPN) for deaf children (Table 2). While the specific developmental changes associated with maturation of the language system, as well as the role of left lateralization in various brain regions, are still under intensive investigation, it is nevertheless well accepted that the representation of language varies with experience and the acquisition of language skills. The relationship between language processing and neural activity may be also different as a function of gender and age. We acknowledge a source of variance within the study population from the inclusion of children and disproportionate numbers of females in the sample, as well as from the wide range in experience and duration of exposure (DOE) to JSL and JPN. All of these factors may limit the generalization of the results to an adult population with typical language development, but it is still challenging to examine whether the functional organization predicted from the previous neuroimaging studies can be demonstrated by such a unique sample of participants. We believe that our participants provide us a rare opportunity to examine the universality of language processing in the brain beyond modality differences and human diversities, and to elucidate the universal relationship between the underlying neural organization and hierarchical linguistic processes.

**Table 1:** **English translation of JSL discourse sentences**.

A:	(a Deaf woman): Next time, would you like to do a “Momo-taro” play?
	(“Momo-taro” is a Japanese folk tale translating as “Peach Boy.”)
B:	(a Deaf man): Let me see … Yes, I would.
A:	Where would you like to do the play?
B:	In the backyard of my house.
A:	Isn't it small for a play?
B:	No, it isn't. It's large enough for 20 people to gather.
A:	Cool! I'm sure to come.
B:	We can watch the play while having BBQ.
A:	That sounds great! Should it be free?
B:	Nonsense! I'll ask a thousand yen including BBQ and the play.
A:	What roles are included in the play?
B:	There are an old man, an old woman, Momo-taro, a dog, a bird, a monkey …
A:	Yeah, the dog, bird, and monkey.
B:	Right. They went to Oni-ga-shima (the “Demons' Island”).
A:	Which won?
B:	Momo-taro won.
A:	Watching the play seems boring to me. BBQ and talking would be more fun.
B:	No, you must take part in the play, too.
A:	No way! I'm too nervous.
B:	Really? For the BBQ, we will have thick, juicy, and delicious steak …
A:	Oh, you're drooling …
B:	Now, will you take part in the play?
A:	OK, I want to take the role of Momo-taro.
B:	No, you should take a Demon, and I'll take Momo-taro.
A:	It's not fair! Let's toss a coin to decide.
B:	Which do you prefer, Momo-taro or a steak?
A:	Wait a minute … a steak.
B:	You can have a steak, if you play a Demon.
A:	All right. Who cares, I'll be a Demon.
B:	Heh-heh-heh.

**Table 2:** **Participants' profiles and behavioral data for the task**.

		**Range**	**Mean**	***SD***
Age (years old)		12–54	30	14
LQ		50–100	93	14
Age of hearing loss (years old)		0–3	0.5	1.0
AOA (years old)	JSL	0–22	8.7	7.3
	JPN	0–18	4.2	4.6
DOE (years)	JSL	1–54	21	17
	JPN	6–50	26	13
Hit rate	Word	0.20–1.00	0.78	0.22
	Sent	0.10–1.00	0.57	0.23
	Disc	0.50–1.00	0.76	0.12
	R	0.78–1.00	0.97	0.05
Correct rejection rate	Word	0.84–1.00	0.97	0.05
	Sent	0.79–1.00	0.94	0.07
	Disc	0.79–1.00	0.91	0.05
	R	0.97–1.00	1.00	0.01
*d*′	Word	0.16–3.58	2.63	0.84
	Sent	0.16–3.26	1.78	0.75
	Disc	1.34–3.81	2.18	0.54
	R	3.24–4.76	4.44	0.44
RTs (ms)	Word	5857–11753	7747	1097
	Sent	4223–9472	6696	902
	Disc	4714–7890	5910	703
	R	543–4802	2549	1180

LQ, laterality quotients; AOA, age of acquisition; DOE, duration of exposure; RTs, reaction times; JSL, Japanese Sign Language; JPN, Japanese; Word, word-level decision; Sent, sentence-level decision; Disc, discourse-level decision; R, repetition.

A recent functional magnetic resonance imaging (fMRI) study of Deaf adults reported that activations in the left frontal and some other regions during a grammatical judgment task were negatively correlated with the ages of acquisition (AOAs) of American Sign Language (ASL) (Mayberry et al., 2011). Because AOAs were also negatively correlated with the task performances in this previous study, it is critical to separate the effects of proficiency and AOAs, and to confirm common activations among all participants. For our functional analyses, we tried to exclude the effects of the following factors by regarding them as nuisance factors: the effect of age, handedness, gender, age of hearing loss, AOAs of JSL, and AOAs of JPN. On the other hand, some previous voxel-based morphometry (VBM) studies have shown that Deaf individuals have an increased gray matter (GM) density in the left motor cortex (Penhune et al., 2003) and an increased GM volume in the insula (Allen et al., 2008), compared with hearing non-signers. It has also been shown that AOAs of ASL were negatively correlated with the GM density in the left precuneus (Pénicaud et al., 2013). We thus hypothesized that there would be an anatomical signature for the linguistic proficiency of individuals in the frontal language areas and these regions, even when the functional organization of the frontal language areas was common to individuals.

## Materials and methods

### Participants

Thirty-seven Deaf signers participated in the present study. Nine participants were excluded from the data analyses because of neurological abnormalities, excessive head movements even in a single run, weak left-handedness, later onset of hearing loss (more than 3 years old), and/or poor task performances (null hit rates). Table 2 shows detailed profiles of the remaining 28 participants (20 females and 8 males), including two students from a bilingual-bicultural school for the Deaf (Meisei Gakuen, Shinagawa-ku, Japan) and three students from a facility for Deaf children (Kanamachi Gakuen, Katsushika-ku, Japan). All participants showed right-handedness [laterality quotients (LQ) = 50], as determined by the Edinburgh inventory (Oldfield, 1971). According to audiological reports, all participants had binaural hearing losses of >75 dB. Most of the 28 participants had experienced the cued speech method and/or the oral method to learn JPN, but there has been no established testing in JSL or JPN for the Deaf. Neither the grouping of early/late bilinguals nor a binary distinction between native/non-native speakers (i.e., first/second languages) was taken into account in the present study. Written informed consent was obtained from all participants, as well as from their parents/guardians for the juvenile participants, according to the Declaration of Helsinki. The study was approved by both the school and facility, and by the review board of The University of Tokyo, Komaba.

### Stimuli

A dialog between two Deaf signers (a woman and a man) was prepared in JSL (see Table 1 for the entire dialog). Each stimulus consisted of familiar signs, which were sufficiently easy for the juvenile participants to comprehend, and did not contain fingerspellings. Each dialog sentence was articulated by one of two signers who were taking turns, i.e., one person questioning/proposing, then the other responding, thereby completing one or two sentences (Figure 1A). The rationale for using two signers was to present samples of natural discourse with rich prosodic cues, thereby providing contextual information through an actual conversation (Sakai et al., 2005). Video-taped signers were always in a full-face shot, because gaze directions are crucial in sign languages. For example, the video images of an inquiring facial expression looking forward represented an interrogative to the other signer in the dialog. Video images of the two signers were presented with an eyeglass-like MRI-compatible display (VisuaStim XGA; Resonance Technology, Northridge, CA) (resolution = 720 × 480, frame rate = 30 fps).

### Tasks

There were three language tasks: word-level or lexical (Word), sentence-level (Sent), and discourse-level (Disc) decision tasks. In the Word and Sent tasks, each dialog sentence was presented in a random order; in the Disc task, the dialog sentences were presented in the original order, and completed in seven separate blocks. Therefore, the overall stimuli were physically equated among the language tasks. On the other hand, individual tasks imposed different “task sets” (i.e., an effective intention for a task to attend the specific operations demanded by the task) as explained below, while these tasks basically included probe detection in common in that anomalous probes *infrequently* appeared (in 10 out of 29 or 43 dialog sentences). It should be noted that there was only one type of probes (lexical, syntactic, or contextual errors) included in each task block. The purpose of including probes was to ensure the participants' full attention to lexical information, sentence expressions, or discourse flows, thereby allowing assessment of their linguistic proficiency for each task. A number of linguistic studies with lexical, syntactic, or contextual decision tasks inherently involved anomaly detection in the tasks, and we have already established that activations of the frontal language areas depend on the type of decisions, but not on the anomaly of stimuli themselves (Suzuki and Sakai, 2003). We performed fMRI experiments in a block design, which measured overall responses during each block, and thus was unaffected by the presence of probes (mostly less than 5 s). Trials with the sentences containing anomalous probes were included in the block design analysis. To maintain the natural flow of signs, both normal and anomalous versions of an entire sentence were filmed with the same signers and settings, as if each sentence constituted a normal continuous discourse even with a probe. Each block of the language tasks consisted of four or five dialog sentences, and lasted for 24–48 s.

Using both JSL and written JPN, we instructed the participants to respond to a probe by pressing a button while a sentence containing the probe was presented. At the initiation of each block, the task type was visually presented for 1.3 s in Japanese: “*kotoba*” (“*word*”) for the Word, “*hyougen*” (“*expression*”) for the Sent, and “*kaiwa*” (“*conversation*”) for the Disc task. In the Word task, probes were pseudo-signs freely devised by the native signers, and the participants were asked to detect the probes by focusing on word-level information among the disconnected dialog sentences. Lexical decision critically involves word-level integration; for sign languages, elements of handshape, location, and movement are combined into real words. In the Sent task, probes were syntactically anomalous JSL expressions, and the participants were asked to detect the probes by focusing on sentence expressions (e.g., word-to-word relationships) among the disconnected dialog sentences. These syntactic errors included violations of tense (see Figure 1A), person (e.g., an agreement error between first and third persons), word order (an ungrammatical order of lexical items), etc. In the Disc task, probes were contextually anomalous signs in the flow of the dialog, and the participants were asked to detect the probes by focusing on the flow of discourse, i.e., using discourse-level integration. Every sentence containing a probe in the Disc task was not only syntactically normal, but also *semantically plausible*, if the sentence was free from a given context. In our paradigm, the levels of linguistic integration necessary for each language task can be characterized by distinct *linguistic units*. In the Word, Sent, and Disc tasks, linguistic units were individual words, disconnected dialog sentences, and consecutive sentences, respectively.

As a baseline for the language tasks, a repetition (R) task was tested with the same probe detection. At the initiation of each R block, the task type, “*kurikaeshi*” (“*repetition*”), was visually presented for 1.3 s. In the R task, normal sentences used in the three language tasks were played backward and presented in a randomized order, and a probe was a successively repeated “backward sentence.” During each R block, video images of only one signer (e.g., a woman for an R block, a man for the next R block, etc.) were presented, since successive presentation of different signers could not become a probe. The participants reported that it was impossible to comprehend sentences composed of backward signs, although some of the signs were recognizable as meaningful. Here, it should be noted that the stimuli used in the language and R tasks were physically equivalent, i.e., in terms of the visual stimuli themselves, which included hand shapes, facial expressions, and body movements. Activation by the contrasts between the language and R tasks (e.g., Word − R) thus reflected the processes of individual words (in common for the language tasks) and associated linguistic integration (different among the language tasks), while general cognitive factors such as lower-level visual perception, probe detection, short-term memory, response selection, and motor responses were fully controlled.

Every participant underwent a total of eight scanning sessions, each of which had seven blocks and lasted for 220.7–221.1 s. In the first seven sessions, four blocks of the baseline R task were alternately presented with a block of the Word, Sent, or Disc task (appearing once in a pseudo-random order). In the last session, four blocks of the R and three blocks of the Disc task were alternately presented, with a part of the original dialog being repeated once more with new probes in the Disc task. In each block of the Disc and R tasks, there was always a single probe, and in each block of the Word and Sent tasks, there were either one or two probes. There were a total of ten different probes for each of the Word, Sent, and Disc tasks, whereas there were 32 probes for the R task. In each language task, no sentence appeared more than twice. The use of more blocks for the Disc task might have increased the brain activations, but the number of blocks was largest for the R task (Figure 1B). Since the number of blocks was equal for the Word and Sent tasks, the change in activation by the levels of linguistic integration, if any, cannot be explained by the differences in the number of blocks.

The stimulus presentation and button-press signal acquisition were controlled using the Lab-VIEW software package and interface (National Instruments, Austin, TX). The accuracy of each task was evaluated with *d*′, which was computed from the *Z*-scores of hit rates and correct rejection rates. If these rates were 1.00, the formula of 1 – 1/(2*N*) was used (Macmillan and Creelman, 2005), where *N* was the total number of probes in each task (Word, 10; Sent, 10; Disc, 10; R, 32) for hit rates, and that of other stimuli in each task (Word, 19; Sent, 19; Disc, 33; R, 110) for correct rejection rates. Reaction times (RTs) were calculated from the onset of each dialog sentence containing a probe.

### MRI data acquisition

The participants were in a supine position, and their heads were immobilized inside the radio-frequency coil with straps. The MRI scans were conducted on a 3.0 T MRI system (GE Signa HDxt 3.0T; GE Healthcare, Milwaukee, WI). We scanned 32 axial slices that were 3-mm thick with a 0.3-mm gap, covering from −42.9 to 62.7 mm from the anterior to posterior commissure (AC-PC) line in the vertical direction, using a gradient-echo echo-planar imaging (EPI) sequence [repetition time (TR) = 3 s, echo time (TE) = 60 ms, flip angle (FA) = 90°, field of view (FOV) = 192 × 192 mm^2^, resolution = 3 × 3 mm^2^]. In a single scanning run, we obtained 77 volumes following three dummy images, which allowed for the rise of the MR signals. High-resolution T1-weighted images of the whole brain (192 axial slices, 1.0 × 1.0 × 1.0 mm^2^) were acquired with a three-dimensional fast spoiled gradient-echo (3D FSPGR) sequence (TR = 9 ms, TE = 3 ms, FA = 25°, FOV = 256 × 256 mm). These structural images were used for normalizing fMRI and VBM data.

### fMRI analyses

We performed group analyses with statistical parametric mapping software (SPM8; Wellcome Trust Center for Neuroimaging, London, UK) run on MATLAB software (MathWorks, Natick, MA). The acquisition timing of each slice was corrected using the middle (sixteenth in time) slice as a reference. We realigned the functional volumes to the first volume and removed runs that included data with a translation of >4 mm in any of the three directions and with a rotation of >2.5°. Each individual's structural image was coregistered to the mean functional image generated during realignment. The coregistered structural image was spatially normalized to the standard brain space as defined by the Montreal Neurological Institute (MNI) using the unified segmentation algorithm with very light regularization, which is a generative model that combines tissue segmentation, bias correction, and spatial normalization in the inversion of a single unified model (Ashburner and Friston, 2005). After spatial normalization, the resultant deformation field was applied to the realigned functional imaging data, which was resampled every 3 mm using seventh-degree B-spline interpolation. All normalized functional images were then smoothed by using an isotropic Gaussian kernel of 9 mm full-width at half maximum (FWHM). Task-specific effects were estimated with a general linear model (random effects model).

In the first-level analysis, each participant's hemodynamic responses induced by the tasks were modeled with a box-car function, and this function was convolved with a hemodynamic function. To minimize the effect of head movements, the six realignment parameters obtained from preprocessing were included as a nuisance factor in a general linear model (Figure 1B). The images of the Disc, Sent, Word, and R tasks were then generated for each participant, and used for a second-level analysis. To regress out the effect of age, LQ, gender, age of hearing loss, AOAs of JSL, and AOAs of JPN, we included these nuisance factors as covariates in the design matrix of the second-level analysis. The statistical parametric maps were thresholded at a voxel level of uncorrected *p* < 0.0001, and at a cluster level of corrected *p* < 0.05 using the false discovery rate (FDR). For the anatomical identification of activated regions, we used the Anatomical Automatic Labeling method (Tzourio-Mazoyer et al., 2002) and Anatomy toolbox (Eickhoff et al., 2005).

### VBM analyses

VBM analyses on MR images were performed using SPM8 software. After alignment to the AC-PC line, T1-weighted images were bias-corrected and segmented to the GM, white matter, and cerebrospinal fluid by using default tissue probability maps and a New Segment tool, which uses an affine regularization to warp images to the International Consortium for Brain Mapping (ICBM) East Asian brain template. Inter-subject registration was achieved with Diffeomorphic Anatomical Registration Through Exponentiated Lie Algebra (DARTEL) (Ashburner, 2007). Jacobian-scaled (“modulated”) and warped tissue class images were then created with DARTEL's Normalize to MNI Space tool, which spatially normalized images to the MNI space, converted voxel sizes to 1.5 × 1.5 × 1.5 mm^3^ (the size of the DARTEL template), and smoothed images with a standard Gaussian filter of 8-mm FWHM. To avoid possible edge effects (partial volume effects) around the border of the GM, voxels with a value greater than 0.2 were used for analyzing the modulated GM images. A multiple regression analysis was performed on the smoothed GM images to determine regions in which GM volumes showed a correlation with the *d*′ value of each language task. The total GM volumes of individual brains were entered into the model as a proportional scaling factor to regress out the general size difference across the participants. The *d*′ of the R task, as well as nuisance factors used in the fMRI analyses, was included as a covariate in the design matrix of VBM analyses. The statistical parametric maps of GM volumes were thresholded at a voxel level of uncorrected *p* < 0.001, and at a cluster level of FDR-corrected *p* < 0.05. To account for the non-isotropic smoothness of the VBM data (Ashburner and Friston, 2000), non-stationary cluster correction implemented in SPM8 was applied.

## Results

### Behavioral data

The task performances were well above the chance level, as indicated by the fact that the value of *d*′ was significantly larger than zero (all, *p* < 0.0001) (Table 2). These high task performances suggest that the participants successfully detected different types of probes. According to a One-Way repeated measures analysis of variance (rANOVA) on the *d*′ data, there was a significant main effect of the task [*F*_(3, 81)_ = 129, *p* < 0.0001]. Paired *t*-tests showed that the R task was significantly easier than the other three tasks (Bonferroni corrected *p* < 0.05). Based on a comparison of the *d*′ values for the three language tasks, the Word task was the easiest, whereas the Sent task was the most difficult (corrected *p* < 0.05).

Correlation analyses on *d*′ among the participants showed that the performance of the Word task was significantly correlated with that of the Sent task (*r* = 0.61, *p* = 0.0004) (Table 3). If some participants had inadvertently and partially switched between the Sent and Word tasks irrespective of task instructions, then the performances of one of the two tasks would have become worse, because the use of one task set, e.g., the detection of pseudo-signs, did not help in the proper use of the other task set, as there were no pseudo-signs at all in the Sent task. However, the performances on the Word task were found to be positively correlated with those on the Sent task, indicating that the participants, who discriminated one of the two tasks well, could also discriminate the other task, even when the same set of disconnected sentences was used.

**Table 3:** **Correlation matrix of participants' profiles and behavioral data**.

	**Age**	**AOA (JSL)**	**DOE (JSL)**	***d*′ (Word)**	***d*′ (Sent)**	***d*′ (Disc)**	***d*′ (R)**
Age	1.00	−0.25	0.91^*^	−0.18	−0.06	0.02	−0.03
AOA (JSL)		1.00	−0.62^*^	0.04	0.22	−0.05	0.15
DOE (JSL)			1.00	−0.16	−0.14	0.00	−0.08
*d*′(Word)				1.00	0.61^*^	0.40	0.29
*d*′(Sent)					1.00	0.29	0.17
*d*′(Disc)						1.00	−0.03
*d*′(R)							1.00

Correlation coefficients (r) are shown here. Asterisks denote significant correlations (corrected p < 0.05).

On the other hand, the ages of the participants were strongly correlated with the DOE of JSL (*r* = 0.91, *p* < 0.0001); the ages and DOE of JPN were also correlated (*r* = 0.94, *p* < 0.0001). The AOAs and DOE of JSL were negatively correlated with each other (*r* = −0.62, *p* = 0.0003), while the AOAs and DOE of JPN were not correlated (*r* = 0.08, *p* = 0.68), probably due to the smaller variances for the AOAs of JPN. The correlation between the AOAs of JSL and JPN was not significant (*r* = −0.22, *p* = 0.28), indicating that these AOAs were independent of each other for the participants.

According to a One-Way rANOVA on the RTs, there was a significant main effect of the task [*F*_(3, 81)_ = 165, *p* < 0.0001]. Paired *t*-tests showed that the RTs for the R task were significantly shorter than those for the other three tasks (corrected *p* < 0.05). Among the RTs for the three language tasks, the RTs for the Word task were the longest, whereas those for the Disc task were the shortest (corrected *p* < 0.05). In the VBM analyses, we used the *d*′ of each task to represent the linguistic proficiency of individuals, because *d*′ controls for any response bias the participants may have, and thus is more reliable than RTs in this context.

### Cortical activations modulated by the levels of linguistic integration

**Figure 2:**
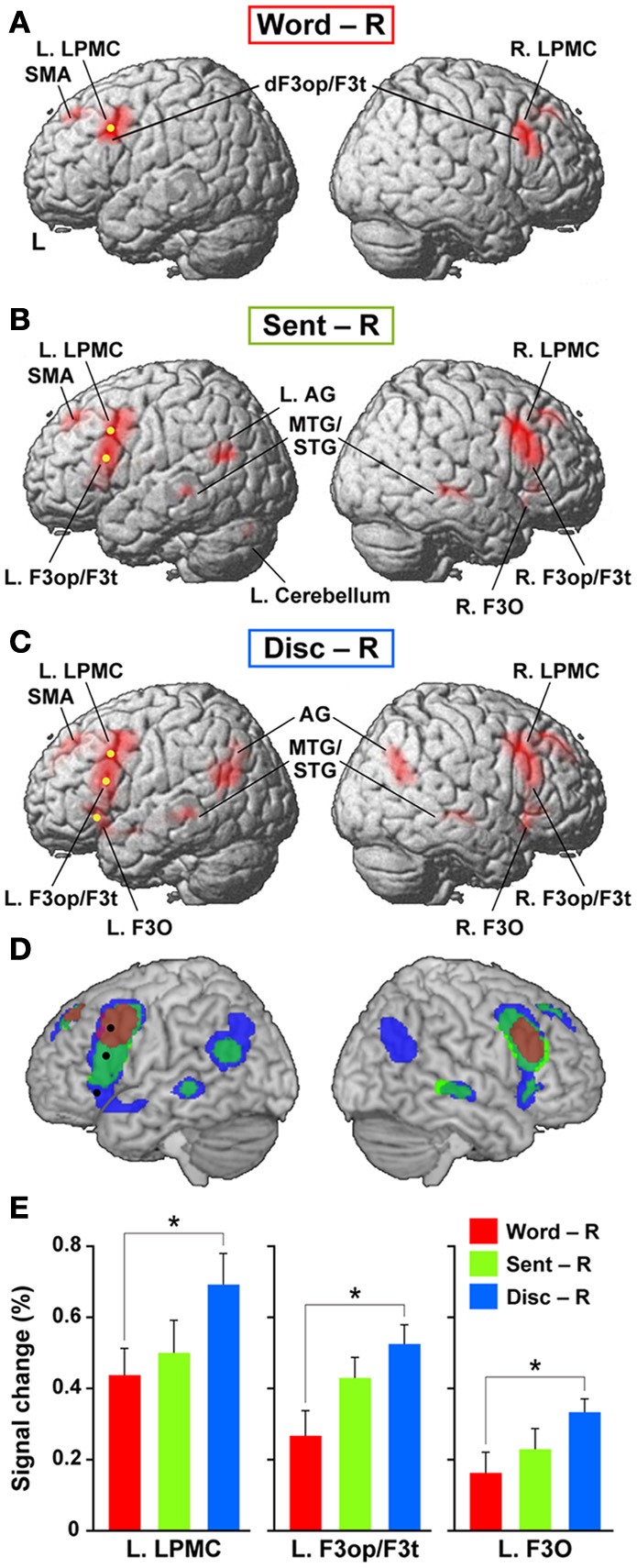
**Cortical activations modulated by the levels of linguistic integration. (A–C)** Cortical activations in each of the Word **(A)**, Sent **(B)**, and Disc **(C)** tasks, compared with the R task, are projected onto the lateral surfaces of a standard brain in the MNI space. Significantly activated regions are shown in red (FDR-corrected *p* < 0.05). Note the most prominent activation in the frontal language areas. **(D)** An overlaid map of cortical activations in Word − R (red), Sent − R (light green), and Disc − R (blue), using transparent overlays in this order (Word − R topmost). For example, when a region was activated in both Sent − R and Disc − R, its color became blue-green (see the region in the right temporal cortex). Note the gradual expansion of activation from the dorsal to ventral regions within the frontal language areas. We focused on three regions in the frontal language areas: the L. LPMC, L. F3op/F3t, and L. F3O [shown as yellow dots in **(A–C)**, and as black dots in **(D)**]. **(E)** Signal changes of each local maximum for Word − R (red), Sent − R (light green), and Disc − R (blue). Error bars indicate the SEM of participants, and asterisks denote the significance level of corrected *p* < 0.05.

To elucidate the functional organization of the frontal language areas in an unbiased manner with respect to other cortical regions, we adopted whole brain analyses for fMRI and VBM. Figures 2A–C show cortical responses in the Word, Sent, and Disc tasks compared with those in the R task (FDR-corrected *p* < 0.05). Corresponding to a difference in the linguistic units for the Word, Sent, and Disc tasks (see Materials and Methods, Tasks), activated regions in the frontal language areas gradually expanded in the dorso-ventral axis, i.e., from the L. LPMC to the L. F3O, via the L. F3op/F3t. In Word − R, significant activation was observed bilaterally in the LPMC and dorsal F3op/F3t (dF3op/F3t), as well as in the pre-supplementary motor area (pre-SMA, the medial side of BAs 6/8) (Table 4). In Sent − R and Disc − R, consistent activation was observed in the bilateral LPMC/F3op/F3t, R. F3O, pre-SMA, left angular gyrus (L. AG, BA 39), bilateral middle/superior temporal gyrus (MTG/STG, BAs 21/22), and bilateral caudate. In Disc − R, activation in the F3O and AG became bilateral, indicating that the Disc task recruited exactly mirrored regions. We also observed the left cerebellum activation in Sent − R, and the medial precuneus (BA 7) activation in Disc − R. Taking these results together in an overlaid map (Figure 2D), the hierarchical integration in the frontal language areas was striking, such that the more dorsal regions activated at the lower levels of linguistic integration were almost completely included in the wider regions activated at the higher levels. In the right lateral frontal cortex, pre-SMA, and right temporal regions, in contrast, such an integration was unclear, and some regions activated at the lower levels were outside those activated at the higher levels.

**Table 4:** **Cortical activations modulated by the levels of linguistic integration**.

**Brain region**	**BA**	**Side**	***x***	***y***	***z***	***Z* value**	**Voxels**
**WORD − R**							
LPMC/dF3op/F3t	6/8/44/45	L	−48	17	37	5.1	224
LPMC/dF3op/F3t	6/8/44/45	R	48	20	31	4.9	136
pre-SMA	6/8	M	−9	44	46	5.0	143
**SENT − R**							
LPMC	6/8	L	−39	11	43	5.5	538
F3op/F3t	44/45	L	−51	20	19	5.2	*
LPMC	6/8	R	39	14	37	6.2	437
F3op/F3t	44/45	R	45	29	19	5.7	*
F3O	47	R	48	26	1	4.2	47
pre-SMA	6/8	M	−9	38	46	6.2	323
AG	39	L	−42	−58	25	4.8	139
MTG/STG	21/22	L	−60	−34	−2	4.6	36
		R	48	−28	−5	5.5	103
caudate		L	−15	5	16	4.6	80
		R	12	5	13	4.5	77
cerebellum, crus I		L	−18	−76	−32	4.9	34
**DISC − R**							
LPMC	6/8	L	−39	8	43	6.6	966
F3op/F3t	44/45	L	−51	20	22	6.5	*
F3O	47	L	−30	26	−5	6.5	*
MTG/STG	21/22	L	−48	−31	−5	6.3	*
LPMC	6/8	R	42	17	43	6.2	577
F3op/F3t	44/45	R	54	23	16	5.6	*
F3O	47	R	36	26	−8	6.1	*
MTG/STG	21/22	R	54	−25	−5	5.6	79
pre-SMA	6/8	M	0	35	46	6.9	562
AG	39	L	−33	−70	40	5.6	333
		R	57	−64	22	5.5	195
precuneus	7	M	−3	−67	31	5.1	93
caudate		L	−15	11	10	5.2	99
		R	15	17	7	4.8	103

Stereotactic coordinates (x, y, z) in the MNI space (mm) are shown for each activation peak of Z values (corrected p < 0.05). BA, Brodmann's area; L, left hemisphere; R (in the Side column), right hemisphere; M, medial; LPMC, lateral premotor cortex; dF3op/F3t, pars opercularis and triangularis of the dorsal inferior frontal gyrus; F3O, pars orbitalis of the inferior frontal gyrus; pre-SMA, pre-supplementary motor area; AG, angular gyrus; MTG/STG, middle/superior temporal gyrus. The region with an asterisk is included within the same cluster shown one row above.

We focused on the frontal language areas with clear hierarchical integration, and chose three regions of interest (ROIs). The local maximum of each region of the L. LPMC, L. F3op/F3t, and L. F3O was taken serially from significant activation in Word − R, Sent − R, and Disc − R, respectively (Table 4), to ensure an unbiased selection of local maxima (i.e., not necessarily selective to Disc − R alone). We examined whether activations of these ROIs were incrementally modulated with the level of linguistic integration (Figure 2E). A Two-Way rANOVA with the ROI (L. LPMC, L. F3op/F3t, L. F3O) × task (Word, Sent, Disc) showed significant main effects of ROI [*F*_(2, 54)_ = 14, *p* < 0.0001] and task [*F*_(2, 54)_ = 8.2, *p* = 0.0008] with no significant interaction [*F*_(4, 108)_ = 1.0, *p* = 0.39]. Paired *t*-tests showed that the signal changes for Disc − R were significantly higher than those for Word − R in all three regions (Bonferroni corrected *p* < 0.05). Task difficulty cannot explain these modulation patterns, as the Sent task was the hardest among the three language tasks; the patterns were also independent of general short-term memory, because we subtracted responses in the R task. Therefore, not only the dorso-ventral expansion of activated regions in the frontal language areas, but also overactivation in each of these regions was primarily influenced by the level of linguistic integration.

### Lateralization and functional localization of cortical activations

**Figure 3:**
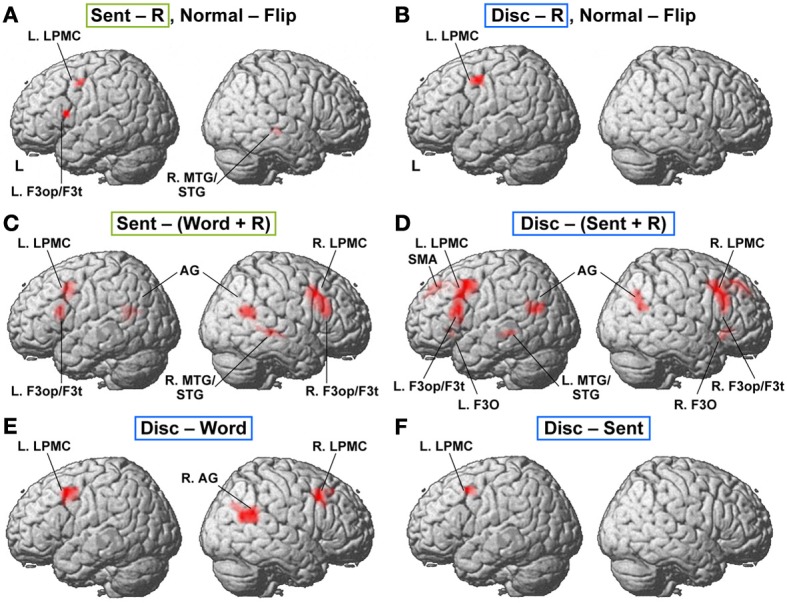
**Lateralization and functional localization of cortical activations. (A,B)** Cortical activations in the Sent − R or Disc − R of the brain images flipped from side to side (Flip) were subtracted from those of the normal images (Normal). In Sent − R, Normal − Flip **(A)**, note the significant effect of hemispheres in the L. LPMC, F3op/F3t, and R. MTG/STG. In Disc − R, Normal − Flip **(B)**, there was a significant effect of hemispheres in the L. LPMC alone. **(C)** Regions identified by Sent − (Word + R). **(D)** Regions identified by Disc − (Sent + R). **(E)** Regions identified by Disc − Word. **(F)** Regions identified by Disc − Sent.

To determine which regions showed significant lateralization, we further performed a flip method with voxel-wise analyses, which has been shown to be superior to the ROI-based lateralization indices method for such determination (Baciu et al., 2005). For this purpose, activations (i.e., in Word − R, Sent − R, or Disc − R) of the brain images flipped from side to side (i.e., mirror-reversed images derived from fMRI first-level analyses) were subtracted from the cortical activations of the normal images shown in Figures 2A–C. These comparisons correspond to the interaction of task and hemisphere (left vs. right); the resultant activations in the left brain represent (Disc − R) × (left − right), etc., whereas those in the right brain represent (Disc − R) × (right − left), etc. (FDR-corrected *p* < 0.05). In Sent − R, we observed clear left-lateralized activations in the L. F3op/F3t and L. LPMC, as well as right-lateralized activations in the MTG/STG (Figure 3A, Table 5). In Disc − R, we observed clear left-lateralized activations in the L. LPMC alone (Figure 3B), while there was no significant lateralization of activations in Word − R.

**Table 5:** **Lateralization and functional localization of cortical activations**.

**Brain region**	**BA**	**Side**	***x***	***y***	***z***	***Z* value**	**Voxels**
**SENT − R, NORMAL − FLIP**							
LPMC	6/8	L	−42	−4	52	4.2	25
F3op/F3t	44/45	L	−51	14	13	4.5	21
MTG/STG	21/22	R	39	−28	−5	4.6	31
**DISC − R, NORMAL − FLIP**							
LPMC	6/8	L	−45	2	46	4.9	56
**SENT − (WORD + R)**							
LPMC	6/8	L	−42	11	43	5.0	208
F3op/F3t	44/45	L	−51	20	19	4.5	*
LPMC	6/8	R	39	11	37	6.1	342
F3op/F3t	44/45	R	45	26	19	5.4	*
AG	39	L	−42	−49	16	4.9	103
		R	57	−58	19	5.7	151
MTG/STG	21/22	R	48	−31	−2	5.1	106
**DISC − (SENT + R)**							
LPMC	6/8	L	−42	8	46	6.5	491
F3op/F3t	44/45	L	−57	20	19	5.8	*
LPMC	6/8	R	39	17	46	5.6	290
F3op/F3t	44/45	R	54	23	19	4.6	*
F3O	47	L	−30	26	−2	4.7	38
		R	36	29	−5	5.9	62
pre-SMA	6/8	M	9	29	49	6.0	352
AG	39	L	−45	−64	22	4.6	130
		R	57	−61	22	4.6	114
MTG/STG	21/22	L	−48	−28	−8	5.3	58
**DISC − WORD**							
LPMC	6/8	L	−39	8	46	5.6	159
		R	39	17	46	4.7	151
AG	39	R	57	−64	19	4.9	197
**Disc − Sent**							
LPMC	6/8	L	−36	8	49	4.6	50

The region with an asterisk is included within the same cluster shown one row above.

Next, we compared activations among the language tasks. By adding the Word task to the R task in Sent − R, i.e., Sent − (Word + R), we examined overactivation during sentence-level processes when compared with lower levels. This contrast exhibited more focal activation in the L. LPMC and L. F3op/F3t (Figure 3C, Table 5), consistent with the left-lateralized activation in these regions (Figure 3A). On the other hand, the Disc − (Sent + R) contrast resulted in an activation pattern (Figure 3D) similar to that in Disc − R (Figure 2C), but the L. F3O activation was clearly separated. A direct comparison of activations between the Disc and Word tasks showed significant activation in the bilateral LPMC and R. AG (Figure 3E), while a direct comparison between the Disc and Sent tasks showed focal activation in the L. LPMC (Figure 3F). This L. LPMC activation is consistent with the left-lateralized activation in this region (Figure 3B). These results clarified the functional localization of the L. LPMC, L. F3op/F3t, and L. F3O.

### Positive correlations between individual task performances and GM volumes

**Figure 4:**
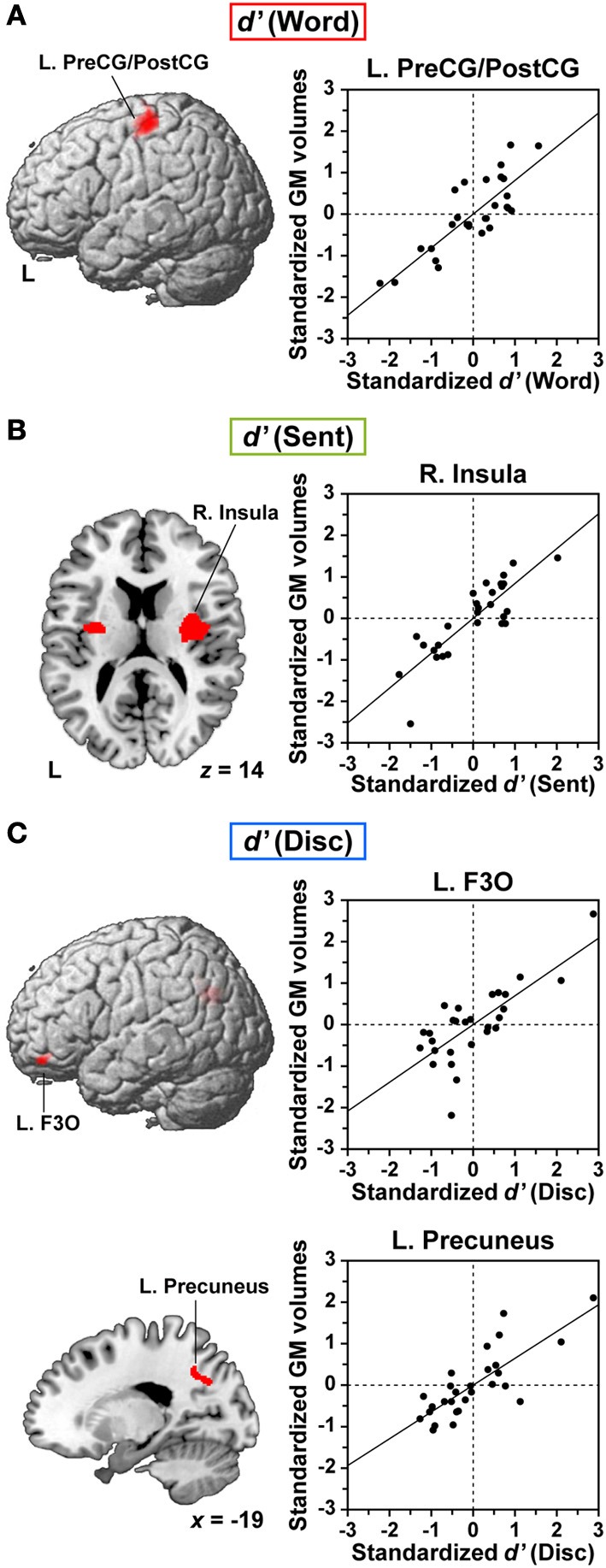
**Anatomical signature for the linguistic proficiency of individuals in JSL. (A–C)** Correlations between regional gray matter (GM) volumes and individual performances (*d*′) of the Word **(A)**, Sent **(B)**, and Disc **(C)** tasks. In the left column, correlation maps (FDR-corrected *p* < 0.05) are projected onto the left lateral surfaces of a standard brain for the L. PreCG/PostCG **(A)** and for the L. F3O **(C)**. An axial slice (*z* = 14) is shown for the largest cluster in the right (R.) insula, as well as for the second largest cluster in the left insula **(B)**. A sagittal slice (*x* = −19) is shown for the largest cluster in the L. precuneus **(C)**. In the right column, scattered plots and regression lines are shown for partial correlations between the standardized *d*′ of each task and the standardized GM volumes at the peak voxel, after removing the effects of age, LQ, gender, age of hearing loss, AOAs of JSL, AOAs of JPN, and *d*′ of the R task.

We further examined correlations between the individual task performances and regional GM volumes. Multiple regression analyses revealed a significantly positive correlation between the *d*′ of each language task and GM volumes in localized regions (FDR-corrected *p* < 0.05). Between the *d*′ of the Word task and GM volumes, we found a prominent correlation in the dorsolateral surface of the left precentral gyrus and postcentral gyrus (L. PreCG/PostCG, BAs 4/3/1/2) [MNI coordinates of its peak: (*x, y, z*) = (−49, −22, 59), *Z*_(19)_ = 4.6, *p* < 0.001, 1227 voxels] (Figure 4A). This region corresponded to the “hand area” of the primary motor and somatosensory cortices. Between the *d*′ of the Sent task and GM volumes, a significant correlation was observed in the right insula [(32, −6, 17), *Z*_(19)_ = 4.9, *p* < 0.001, 1440 voxels] (Figure 4B). The second largest cluster was located in the left insula [(−29, −9, 8), *Z*_(19)_ = 3.9, *p* < 0.001, 449 voxels], which was just below the threshold of FDR-corrected *p* < 0.05. Finally, we also found a strong correlation between the *d*′ of the Disc task and GM volumes in the L. F3O [(−46, 36, −24), *Z*_(19)_ = 4.0, *p* < 0.001, 593 voxels] and in the left precuneus [(−19, −54, 41), *Z*_(19)_ = 4.2, *p* < 0.001, 590 voxels] (Figure 4C). The L. F3O cluster was anteroinferior to the region identified in the Disc − R (Figure 2C), and the left precuneus cluster was lateral to the region identified in the Disc − R. These results suggest an anatomical signature for the linguistic proficiency of individuals in a task-dependent manner.

## Discussion

Here we analyzed cortical activity and GM volumes of Deaf participants, and clarified three major points. First, we found that activated regions in the frontal language areas gradually expanded in the dorso-ventral axis, corresponding to a difference in linguistic units for the three language tasks. Moreover, activations in each region of the frontal language areas were incrementally modulated with the level of linguistic integration. These dual mechanisms of the frontal language areas may reflect a basic organization principle of hierarchically integrating linguistic information. A previous fMRI study with passive (i.e., without on-line tasks) reading of English texts has reported that activations in the bilateral frontal and temporal regions increased in magnitude and spatial extent for each of these regions, with the greatest increase being induced by narratives, followed in order by unconnected sentences and word lists (Xu et al., 2005). However, neither hierarchical integration nor activation modulation by the linguistic levels free from stimulus differences has been previously clarified. We experimentally manipulated the task sets and linguistic units, separately from the stimuli themselves (see Materials and Methods, Tasks), and clarified that active integration processes actually modulated activations in the frontal language areas. Secondly, activations in the LPMC and F3op/F3t were left-lateralized, and direct comparisons among the language tasks exhibited more focal activation in these regions and the L. F3O, suggesting their functional localization. Thirdly, we found significantly positive correlations between individual task performances and GM volumes in localized regions, even when the AOAs of JSL and JPN were factored out. More specifically, correlations with the performances of the Word and Sent tasks were found in the L. PreCG/PostCG and insula, respectively, while correlations with those of the Disc task were found in the L. F3O and left precuneus. These correlations suggest *anatomical* specialization of these regions related to individual abilities in sign languages, irrespective of a wide range of AOAs in both sign and vocal/written languages. These results demonstrate functional and anatomical correlates of hierarchical linguistic integration in the frontal language areas.

The present results suggest that the L. LPMC, L. F3op/F3t, and L. F3O may primarily subserve word-, sentence-, and discourse-level integration, respectively. Hagoort and others have proposed a caudo-rostral gradient of the left inferior frontal cortex, in which unification processes of phonological, syntactic, and semantic elements are gradually represented in three corresponding regions from the left ventral BA 6 to BA 47 (Hagoort, 2005; Uddén and Bahlmann, 2012). In our previous fMRI and lesion studies, however, the L. LPMC was more crucially recruited for processing scrambled sentences (grammatical in JPN) than active sentences, even when phonological factors were thoroughly controlled (Kinno et al., 2008, 2009). The overactivation in the L. LPMC in the Disc task compared with the Word and Sent tasks (Figures 3E,F) can be explained by the hierarchical nature of linguistic integration, such that the Disc task with discourse-level integration requires more intensive checking processes at word- and sentence-levels than do the other tasks without discourse-level integration. We propose that the input-driven information integrated at the word-level in the L. LPMC is transmitted to the center of sentence-level integration in the L. F3op/F3t, and that the L. F3O integrates the information from these regions into coherent and meaningful discourses.

The hierarchical integration in the frontal language areas in the present study may be reminiscent of the recently proposed idea of a “temporal receptive window” (TRW) (Hasson et al., 2008; Lerner et al., 2011), which is the time length of effective stimuli for a cortical region, in analogy with the spatial receptive fields of neurons in the visual system (Hubel, 1988). By using an intersubject correlation analysis for fMRI data, Hasson et al. have shown a hierarchical organization of TRWs from lower- to high-order areas within the bilateral occipital and temporo-parietal cortices for silent movies and speech sounds, respectively. In the bilateral frontal cortex, however, responses were scattered and limited to longer TRWs in these previous studies. In the present study, in contrast, hierarchical integration was most prominent in the frontal language areas, and this organization was closely linked with integration of linguistic information, rather than that of sensory information. Moreover, each specific region of the frontal language areas was overactivated at higher levels of linguistic integration (Figure 2E), whereas the regions with shorter TRWs (e.g., the primary visual and early auditory cortices) responded similarly to stimuli regardless of a larger temporal context in their studies. The sources of these distinctions could be the different stimuli, tasks, or analyses employed, as well as the hierarchical nature of linguistic integration. Nevertheless, it would be interesting to imagine that specific linguistic functions take over a general organizing principle based on TRW in the frontal language areas.

Lesion studies have shown that, as in vocal languages, damage in the left hemisphere caused aphasia in sign languages, while body movements or production of non-linguistic gestures were relatively spared (Corina et al., 1992; Hickok et al., 1996; Marshall et al., 2004). Many previous neuroimaging studies have also confirmed the left-lateralized activations in the fronto-parietal or fronto-temporal regions during phonological (MacSweeney et al., 2008), lexical (Leonard et al., 2012), and grammatical (Mayberry et al., 2011) judgment tasks in sign languages, while the spatial aspects of sign languages may activate the right hemisphere (Emmorey et al., 2002; Newman et al., 2002). In our previous fMRI study (Sakai et al., 2005), we examined cortical activity by contrasting similar Disc and Word tasks in JSL, and reported left-lateralized activation in the frontal and temporo-parietal regions in the JSL task for Deaf signers and hearing bilinguals (children of Deaf adults, CODA). By introducing the Sent task in the present study, we further demonstrated that syntax-selective activations in the L. LPMC and L. F3op/F3t were clearly left-lateralized (Figure 3A). Our results establish that some aspects of the functional organization of frontal language areas in word-, sentence-, and discourse-level processing are common to all individuals, even if there is a considerable individual variability in linguistic proficiency.

In addition to the lateral frontal cortex, we observed consistent activation in the pre-SMA in the language tasks (Table 4). The pre-SMA has been associated with motor control for sequential articulations of syllables (Ziegler et al., 1997; Bohland and Guenther, 2006), and with visuo-spatial transformation in tasks such as mental rotation (Ecker et al., 2006), either of which may explain the consistent pre-SMA activation in the present study. In both the Sent − R and Disc − R, we also observed activation in the temporo-parietal regions, including the bilateral MTG/STG and the left AG, which are involved in phonological and lexico-semantic processes (Dronkers et al., 2004; Hickok and Poeppel, 2007). It is possible that these regions are recruited more intensively during the Sent and Disc tasks, i.e., at higher levels of integration of the phonological and lexico-semantic information. On the other hand, the right AG activation was selective to the Disc task (Figures 2D, 3E), indicating its recruitment only at the highest level of linguistic integration. In addition to these cortical activations, we observed significant activation in the cerebellum in Sent − R, and that in the caudate in both Sent − R and Disc − R. Some previous studies have suggested that on-line linguistic processing is also controlled by the internal model of the cerebellum (Ito, 2008; Lesage et al., 2012), and that the caudate plays an important role in bilingual switching (Crinion et al., 2006; Tan et al., 2011). Our task actually required on-line detection of linguistic errors with a time constraint, and most of the participants were bilinguals in JSL and JPN.

Previous VBM studies have shown that increased GM volumes in distinct regions were associated with proficiencies in different aspects of vocal languages, including vocabulary, literacy, or syntactic abilities (Mechelli et al., 2004; Lee et al., 2007; Carreiras et al., 2009; Nauchi and Sakai, 2009). The increased GM volume in the dorsolateral surface of the L. PreCG/PostCG (i.e., the “hand area”) may be related to the better acquisition of subtle and complex hand movements by signers, who would therefore have better lexical knowledge in JSL as well. A previous VBM study has shown that GM volumes in the *bilateral* primary motor and somatosensory cortices were larger in professional keyboard players than non-musicians (Gaser and Schlaug, 2003). It is striking to note that the signers with right-hand dominance for general motor controls in our study showed a prominent correlation in the *left* PreCG/PostCG. Indeed, even two-hand signs have certain phonological constraints, such that the handshapes and movements must be either more complex in the dominant hand or symmetric in both hands (Sandler and Lillo-Martin, 2006). As regards the insula with a significant correlation between its GM volumes and the *d*′ of the Sent task, previous voxel-based lesion symptom mapping and fMRI studies have shown that the anterior insula is critical for coordinating speech articulation (Dronkers, 1996; Bohland and Guenther, 2006; Kemeny et al., 2006; Baldo et al., 2011). The precuneus, in which we observed a significant correlation between the GM volumes and the *d*′ of the Disc task, has been shown to be involved in the shifting attention between different locations in space necessary for the coordination of motor control (Wenderoth et al., 2005), and also to be necessary for the production of signs with both hands. Based on the suggested motor-related functions of the L. PreCG/PostCG, insula, and precuneus, we suspect that these regions have supportive roles in accomplishing correct linguistic decisions in sign languages. In contrast, the L. F3O, the GM volumes of which were significantly correlated with the *d*′ of the Disc task, would also be functionally specialized in discourse-level integration, further supporting its pivotal role in sentence comprehension.

In spite of the fact that we utilized JSL and examined Deaf signers, which included some children and disproportionate numbers of females, we found *consistently* left-lateralized activation in the frontal language areas among the participants we tested (Figures 2, 3). Moreover, each of these activated regions precisely matched one of those reported by the previous neuroimaging studies with vocal languages, which showed the involvement of the L. LPMC and/or the L. F3op/F3t in syntactic processing, as well as that of the left F3O in sentence comprehension (see the Introduction). The new finding of the present study is that the frontal language areas in the dorso-ventral axis are hierarchically organized in correspondence with the different levels of linguistic integration. The striking consistency of the organization within our Deaf population indicates the *universality* of linguistic processing beyond modality differences and human diversities, which would also be generalizable to other individuals in any natural languages that are based on universal grammar (Chomsky, 1995). In conclusion, our study demonstrated the functional and anatomical correlates of hierarchical linguistic integration in the frontal language areas and other regions. The unification of functional and anatomical studies would thus be fruitful for understanding human language systems from the aspects of both universality and individuality.

## Author contributions

Conceived and designed the experiments: Tomoo Inubushi and Kuniyoshi L. Sakai. Performed the experiments: Tomoo Inubushi and Kuniyoshi L. Sakai. Analyzed the data: Tomoo Inubushi and Kuniyoshi L. Sakai. Wrote the paper: Tomoo Inubushi and Kuniyoshi L. Sakai.

### Conflict of interest statement

The authors declare that the research was conducted in the absence of any commercial or financial relationships that could be construed as a potential conflict of interest.
